# An Image Analysis Pipeline for Quantifying the Features of Fluorescently-Labeled Biomolecular Condensates in Cells

**DOI:** 10.3389/fbinf.2022.897238

**Published:** 2022-06-06

**Authors:** David W. Baggett, Anna Medyukhina, Swarnendu Tripathi, Hazheen K. Shirnekhi, Huiyun Wu, Stanley B. Pounds, Khaled Khairy, Richard Kriwacki

**Affiliations:** ^1^ Department of Structural Biology, St. Jude Children’s Research Hospital, Memphis, TN, United States; ^2^ Center for Bioimage Informatics, St. Jude Children’s Research Hospital, Memphis, TN, United States; ^3^ Department of Biostatistics, St. Jude Children’s Research Hospital, Memphis, TN, United States; ^4^ Integrated Biomedical Sciences Program, The University of Tennessee Science Center, Memphis, TN, United States

**Keywords:** fluorescence microscopy, biomolecular condensate, liquid-liquid phase separation, image analysis, puncta features, open-source software

## Abstract

Biomolecular condensates are cellular organelles formed through liquid-liquid phase separation (LLPS) that play critical roles in cellular functions including signaling, transcription, translation, and stress response. Importantly, condensate misregulation is associated with human diseases, including neurodegeneration and cancer among others. When condensate-forming biomolecules are fluorescently-labeled and examined with fluorescence microscopy they appear as illuminated foci, or puncta, in cells. Puncta features such as number, volume, shape, location, and concentration of biomolecular species within them are influenced by the thermodynamics of biomolecular interactions that underlie LLPS. Quantification of puncta features enables evaluation of the thermodynamic driving force for LLPS and facilitates quantitative comparisons of puncta formed under different cellular conditions or by different biomolecules. Our work on nucleoporin 98 (NUP98) fusion oncoproteins (FOs) associated with pediatric leukemia inspired us to develop an objective and reliable computational approach for such analyses. The NUP98-HOXA9 FO forms hundreds of punctate transcriptional condensates in cells, leading to hematopoietic cell transformation and leukemogenesis. To quantify the features of these puncta and derive the associated thermodynamic parameters, we developed a live-cell fluorescence microscopy image processing pipeline based on existing methodologies and open-source tools. The pipeline quantifies the numbers and volumes of puncta and fluorescence intensities of the fluorescently-labeled biomolecule(s) within them and generates reports of their features for hundreds of cells. Using a standard curve of fluorescence intensity versus protein concentration, the pipeline determines the apparent molar concentration of fluorescently-labeled biomolecules within and outside of puncta and calculates the partition coefficient (K_p_) and Gibbs free energy of transfer (ΔG_Tr_), which quantify the favorability of a labeled biomolecule partitioning into puncta. In addition, we provide a library of R functions for statistical analysis of the extracted measurements for certain experimental designs. The source code, analysis notebooks, and test data for the Punctatools pipeline are available on GitHub: https://github.com/stjude/punctatools. Here, we provide a protocol for applying our Punctatools pipeline to extract puncta features from fluorescence microscopy images of cells.

## Introduction

The biomolecular components of cells are compartmentalized within organelles and other structures, wherein they perform their biological functions. While many cellular compartments enclose their components within bilayer membranes, others lack a membrane and are referred to as membrane-less organelles or biomolecular condensates. These membrane-less compartments commonly form in cells through the process of liquid-liquid phase separation (LLPS), through which biomolecules exist within two distinct phases, a so-called “light phase” with low biomolecule concentrations and a “dense phase” wherein biomolecules are more highly concentrated (Flory and Krigbaum, 1951; Banani et al., 2017). Depending upon the relative thermodynamic favorability of homotypic versus heterotypic interactions, LLPS causes the formation of cellular biomolecular condensates highly enriched in a single species or others containing multiple components (Riback et al., 2020). The thermodynamic favorability and kinetics of the multivalent biomolecular interactions that drive LLPS influence the physical features of the resulting condensates, including their size, shape, material properties (e.g., viscosity and surface tension) and extent of partitioning of biomolecules within them, quantified by the partition coefficient (K_p_) (Feric et al., 2016; Mitrea et al., 2016; Riback et al., 2020). Recognition that biomolecules within large portions of the cell interior are organized through LLPS has revolutionized the fields of cell and structural biology.

Liquid-liquid phase separation by biomolecules has been recognized since 2009, first in the context of live cells (Brangwynne et al., 2009) and later in reconstituted systems with purified components (Li et al., 2012). While many experimental techniques are used to study the process of LLPS (Mitrea et al., 2018), fluorescence microscopy of biomolecular condensates containing fluorescently-labeled components has emerged as a primary method. Commonly, proteins are genetically encoded with a fluorescent protein label (e.g., green fluorescent protein, GFP) (Giepmans et al., 2006) and expressed in live cultured cells for studies of biomolecular condensates. Performing confocal fluorescence microscopy in live cells preserves active biological processes and biomolecular dynamics, and enables quantitation of physical features of condensates containing fluorescently-labeled components, including number, size and shape, as well as their material (Feric et al., 2016) and thermodynamic (Riback, et al., 2020) properties. The extent to which proteins partition into condensates is quantified by the partition coefficient (K_p_), mentioned above, which is the ratio of dense phase and light phase concentrations ([DP] and [LP], respectively; K_p_ = [DP]/[LP]). The thermodynamic favorability of protein partitioning into condensates is expressed as the Gibbs free energy of transfer (ΔG_Tr_ = -RT•ln (K_p_)) (Riback et al., 2020). Thus, fluorescence microscopy not only allows quantitation of physical features of biomolecular condensates (e.g., their number and size in cells) but by noting the relative difference of labeled material within and outside of condensates, it can also quantify the driving force underlying their formation through LLPS (ΔG_Tr_). Access to these types of quantitative information obtained through live cell fluorescence imaging enables rigorous comparison of condensates formed through phase separation under different cellular conditions, or by different fluorescently-labeled proteins. Studies that derive these biophysical parameters have been reliant on manual determination of puncta features, performed in simplified systems, or used custom-made scripts to perform analyses. As these approaches are labor-intensive and subject to user error, the field of biological condensates can benefit from automated analysis of fluorescence microscopy images to quantify puncta within cells and extract biophysical parameters.

NUP98 fusion oncoproteins arise through gene translocations that fuse the N-terminal protein coding region of *NUP98* to C-terminal coding regions of > 30 different genes that commonly encode DNA binding or chromatin binding protein domains (Michmerhuizen et al., 2020) and are oncogenic drivers in ∼10% of pediatric cases of acute myeloid leukemia (AML) (Michmerhuizen et al., 2020). NUP98 fusion oncoproteins direct formation of DNA/chromatin-associated condensates, which recent studies (Ahn et al., 2021; Chandra et al., 2022) showed drive aberrant gene expression, transformation of hematopoietic cells, and leukemogenesis. NUP98-HOXA9 (abbreviated NHA9) is a prototypical NUP98 fusion oncoprotein that, when labelled with monomeric EGFP (termed G-NHA9), was visualized using confocal fluorescence microscopy to form hundreds of dense puncta in the nuclei of live cells. We initially applied the 3D object counter plugin of FIJI (Bolte and Cordelieres, 2006) to three-dimensional z stacks of live cell fluorescence microscopy images to obtain features of puncta formed by G-NHA9. However, the outcome of this analysis was poor, and therefore we were motivated to develop a customized image analysis pipeline (termed “Punctatools”). Herein we discuss the development of the Punctatools pipeline and its application to puncta formed by G-NHA9 in HEK293T cells, as well as those formed by mutant forms of G-NHA9. We demonstrate that the pipeline allows accurate segmentation of nuclear puncta formed by the G-NHA9 constructs and extraction of the associated quantitative puncta features. Access to this information enabled us to understand how the different interaction mechanisms contributing to LLPS by G-NHA9 govern puncta formation in cells. Accompanying this manuscript, we provide the computer code associated with the Punctatools image analysis pipeline, as well as scripts for performing standard analyses of puncta features and associated statistical analyses.

## Materials and Equipment

### Reagents and Equipment

Example data were prepared using HEK293T cells (American Type Culture Collection; RRID:CVCL_0063) transfected with three different previously reported monomeric EGFP (mEGFP)-labeled NHA9 constructs, G-NHA9 (the wild-type fusion oncoprotein), G-NHA9-21FGAA (with alanine mutations in 21 FG motifs), and G-NHA9-ΔDNA (with mutations in the HOXA9 homeodomain that abrogate DNA binding) (Chandra et al., 2022). Immediately prior to imaging, cell culture media was replaced with phenol red-free imaging media supplemented with Hoechst dye (Thermofisher: CAT R37605 and 21063045, respectively) to enable visualization of cell nuclei using high-resolution fluorescence microscopy. Imaging was performed using a 3i Marianas microscope system equipped with a 100X objective, 405 nm (for Hoechst dye) and 488 nm (for mEGFP-labeled proteins) laser lines, and Slidebook 6.0 software. Full details on the preparation and imaging of cells, including reagents, procedures, and acquisition methods were reported previously (Chandra et al., 2022). We also generated purified mEGFP and prepared solutions at concentrations ranging from 1 nM to 100 μM; these solutions were individually imaged using the same microscope settings as used for cell imaging. The image results were analyzed and plotted to give a standard curve of the relationships between fluorescence intensity per square pixel in a single *z*-layer image slice and molar mEGFP concentration. These relationships were linear and were used to convert fluorescence intensities within regions of interest (ROIs) and puncta in cell images to molar mEGFP concentrations.

### Software

The image analysis pipeline utilizes Python and is compatible with versions 3.7–3.9 (Van Rossum and Drake, 2009). The analysis relies on use of the Punctatools package developed herein and its dependencies. For instructions on how to install Punctatools, please refer to the package’s Github page https://github.com/stjude/punctatools. All dependencies will be installed automatically when running the installation script. Microscopy images as well as cell and puncta masks were examined using FIJI (Schindelin et al., 2012) (https://imagej.net/software/fiji/). The FIJI package 3D-object counter (Bolte and Cordelieres, 2006) was used to generate independent puncta segmentations for comparison against Punctatools pipeline results. R-studio (R 4.1.0) (R Development Core Team, 2019) was used to plot puncta features, calculate thermodynamic parameters, and perform statistical analysis.

## Methods

**FIGURE 1:**
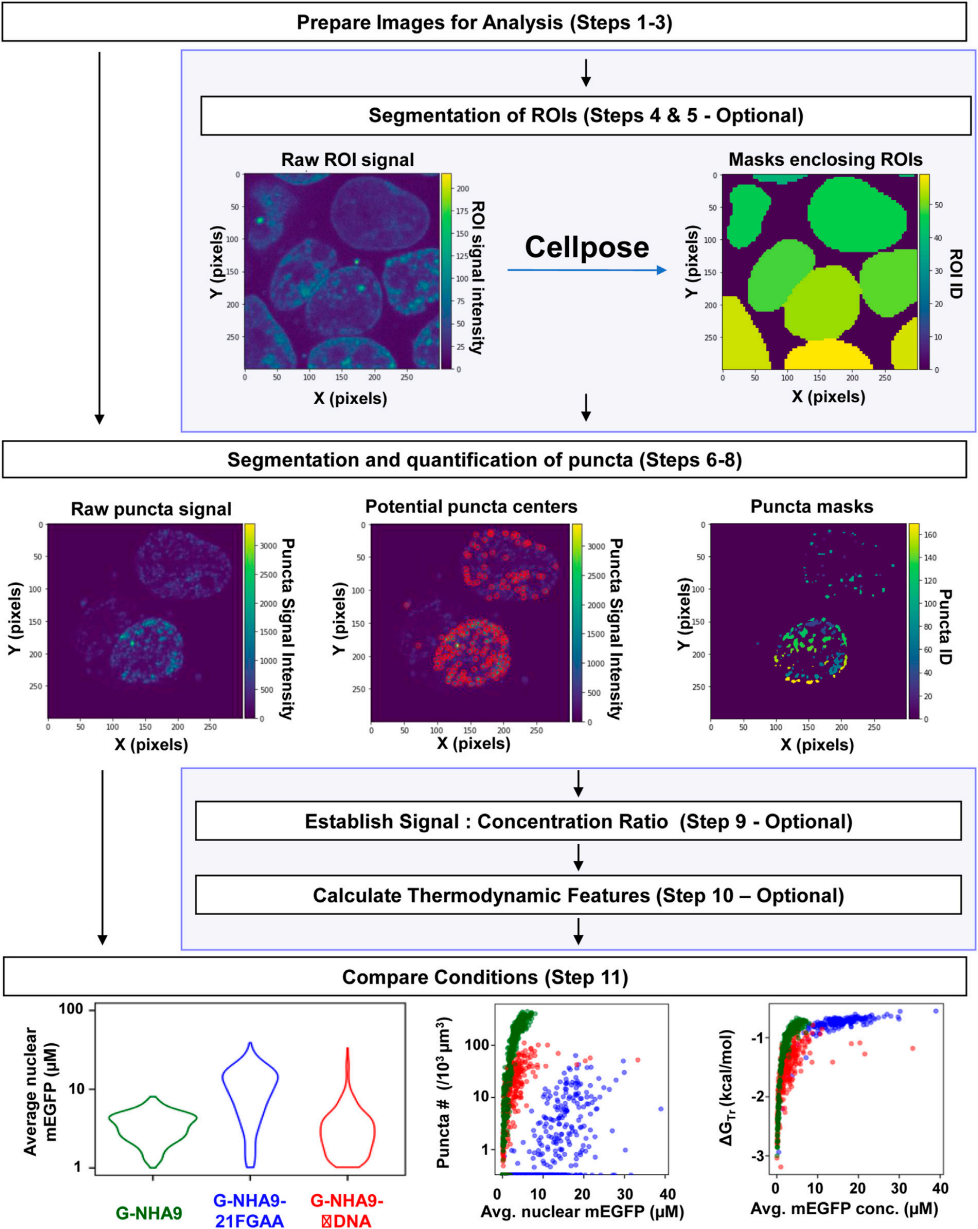
The Punctatools image analysis pipeline. After samples are prepared and image stacks are acquired (Steps 1–3), users are first given the option to segment regions of interest (ROIs) using Cellpose (Steps 4 and 5). Following this, puncta centers are identified from raw puncta signals, and puncta are segmented from the background (Steps 6–8). Users are given the option to establish a signal to concentration ratio (Step 9), which can be used when calculating thermodynamic features (Step 10) and making statistical comparisons (Step 11). Optional steps are highlighted by light-blue background. Average mEGFP concentrations shown on the graph *x*-axes are estimates derived from calibration and are subject to the limitations of this calibration as noted in the main text.

The Punctatools workflow can be divided into four stages (Figure 1): Sample Preparation and Image acquisition (Steps 1–3), Optional ROI Segmentation (Steps 4 and 5), Puncta segmentation and quantification (Steps 6–8) and Results Analysis (Steps 9–11).

### Step 1: Preparation of Samples

The NHA9 fusion oncoproteins used in our puncta imaging studies were fluorescently-labeled at the N-terminus with mEGFP and expressed through transient transfection in HEK293T cells using CL20 backbone plasmids (Chandra et al., 2022). While mEGFP was used in our studies, fluorescent proteins with different spectral properties are available (Giepmans et al., 2006). The G-NHA9 proteins form puncta in the nuclei of HEK293T cells; we used Hoechst dye to fluorescently label DNA and enable segmentation of nuclei. To enable conversion of mEGFP fluorescence intensity values for G-NHA9 proteins within and outside puncta to molar concentrations, we determined a standard fluorescence intensity curve by performing fluorescence microscopy imaging with solutions containing purified mEGFP protein at concentrations ranging from 1 nM to 100 μM (Chandra et al., 2022).

### Step 2: Acquisition of images

Data for the G-NHA9 proteins were collected as 3D z-stacks with pixel size of 0.11 µm in the x- and y-dimensions (total image dimensions were 992 pixels by 992 pixels in the x- and y-dimensions, respectively) and 0.2 µm spacing between planes in the z-dimension, spanning a total z-dimension height of 12 µm. We record 3D z-stacked images at 15 to 20 different positions within the field of view for each condition (e.g., for each of three G-NHA9 protein constructs, wild-type and two mutants). Additional experimental details can be found in our published work (Chandra et al., 2022). The Punctatools pipeline is also capable of analyzing 2D images (a single z-plane), although many of the extracted parameters such as those dealing with volume will have reduced accuracy. Using the above protocol, we obtained images of hundreds of cells displaying fluorescently-labeled nuclear puncta. The number of cell positions that need to be imaged will vary based on transient transfection efficiency and cell confluency. In our studies, we imaged between 50–250 cells that displayed fluorescent signals for each condition to achieve sufficient statistical power for comparisons.

As a guideline, the microscope objective used should be capable of x-, y- and *z*-axis resolutions that are less than the minimum dimensions of the puncta being quantified. Post processing methods such as deconvolution should not prohibit analysis with our pipeline, but care should be taken to account for any artifacts generated by such techniques. We note that the accuracy of data generated by this pipeline will be directly impacted by quality of the microscopy images being analyzed.

### Step 3: Data staging

To process an image and quantify puncta features within, our pipeline requires all of the information for each field of view to be in a single file. If possible, users are encouraged to export data from the microscopy software as a single z-stack including all optical channels. This is the simplest way to prepare data for processing with this pipeline. We have also identified two alternative cases and provide tools to prepare data in the proper format.

If the data is in a format that includes multiple positions or timepoints in a single file (for example, using the Slidebook format), tiff files for individual positions can be extracted using an in-house developed FIJI macro we provide in the distribution. First, create a directory to store the extracted files, then run the conversion macro “export_multipage.ijm” and specify a target location for the new extracted files when asked. The macro will then convert the multi-stack file into individual z-stacks compatible with this pipeline.

If data is formatted as a single image for each z-slice and each channel, it can be reformatted into single z-stacks for each acquisition. To do this, first ensure that the files adhere to a consistent naming convention. File names should include the [SAMPLE_NAME] which denotes a unique acquisition. In addition to the sample name, there should be specific identifiers for the z position (e.g., “_Z”) and channel (e.g., “_C”) that immediately precede the channel and z position numbers, respectively. An example of a properly formatted image name is: “my_sample_position_3_Z00_C01.tif”. Once these files are prepared and properly named, open the “notebooks/setup_images_to_stack.ipynb” notebook and follow the included instructions to adjust the conversion parameters (such as channel code and z-position code). You will have an option to specify pixel size and z-spacing if they are missing from the image metadata. Providing correct values for these parameters is critical for determination of accurate puncta volumes. The output of this notebook will be a (json format text) file with conversion parameters. After creating this parameter file, open the “notebooks/run_images_to_stack.ipynb” notebook, specify the path to the json parameter file generated in the previous step, and run the notebook to convert the entire dataset into compiled tiff files.

Once image input files are properly formatted, they can be divided into folders where each folder denotes a different condition (e.g. “condition1/my_sample_position_3_Z00_C01. tif”). The pipeline will note the name of each folder and include this information in the final output files, allowing for easy comparison between conditions.

### Optional Step 4: Set-Up ROI (Cell/Nuclei) Segmentation

Segmentation of ROIs allows for the analysis and quantification of individual regions such as cells or nuclei. The Punctatools pipeline utilizes Cellpose (Stringer et al., 2021) to segment ROIs using fluorescence signals. To adjust the segmentation parameters, first open the “notebooks/setup_cell_segmentation.ipynb” notebook and follow the instructions to specify the path to the converted dataset, channel of interest, approximate ROI size, and the parameters for the Cellpose algorithm (Table 1). We recommend that users start with default values for segmentation parameters; in most cases these will provide sufficient accuracy for cell/nuclei segmentation. After specifying segmentation parameters, the setup notebook performs an example ROI segmentation that can be used to verify and adjust the parameters (see Table 1 for details). We recommend that users repeat this step for several example images to ensure that segmentation results are consistently satisfactory. Once appropriate parameters have been determined, execute the rest of the notebook, which will save the adjusted parameter values to a json file which is used in subsequent steps.

**TABLE 1:** List of parameters for ROI segmentation (setup_roi_segmentation.ipynb).

Parameter	Definition	Recommended initial value	Notes
Input_dir	Directory with images to be analyzed		All z-layers and channels for a specific sample must be combined into a single file (see Step 3 of the Protocol Procedure).
Output_dir	Directory to save ROI segmentation results		ROI masks will be added as an extra channel to the input image and saved in this directory.
Channel	Channel index, starting from 0, that will be used to segment ROIs		Cellpose allows using nuclei channel to improve whole-cell segmentation. To use this option, provide two channel indices as a list, where the first index corresponds to the nuclei staining, and the second index corresponds to the cytoplasm staining Examples: 0–the first channel will be used to segment ROIs (either cells or nuclei) [1, 0]–the second channel (1) will be used as an auxiliary nuclei stain, the first channel (0) will be used to segment whole cells.
Diameter	Target ROI (cell or nucleus) diameter in pixels		An example image displayed in the notebook will contain scale in pixels to help determine the target ROI diameter. Set to None to automatically detect the ROI diameter.
Model_type	Cellpose model to use for segmentation: ‘nuclei’ for nucleus segmentation, “cyto” or “cyto2” for cell segmentation	cyto	We found that “cyto” and “cyto2” models work better than “nuclei” for segmenting nuclei with irregular shapes.
Gpu	If True, cellpose segmentation will run on GPU; if False, cellpose will use CPU	True	GPU processing is significantly faster; use gpu = True whenever possible.
Do_3D	If True, cellpose segmentation is performed in 3D; if False, cellpose segments ROIs in each individual z-layer, and the ROIs are combined in 3D in the postprocessing	False	3D segmentation is resource intensive, though sometimes more accurate. If do_3D = True results in “CUDA out of memory” error, either set do_3D = False, or set gpu = False.
Flow_threshold	Cellpose parameter: the maximum allowed error of the flows for each mask	0.4	Advanced parameter. Increase if cellpose returns too few masks; decrease if cellpose returns too many ill-shaped masks.
cellprob_threshold	Cellpose parameter: defines which pixels are used to run dynamics and determine masks	0	Advanced parameter. Decrease if cellpose returns too few ROIs; increase if cellpose returns too many ROIs; values should be between -6 and 6.
Remove_small_mode	"2D”, or “3D”. Used to remove small ROIs by volume (3D) or area (2D)	3D	Set to “3D” unless testing on cropped images. Set to “2D” if the image contains only a few z-layers. If set to “3D”, small ROIs are excluded based on volume; this will exclude a ROI if only small part of it is contained in the field of view.
Remove_small_diam_fraction	Size threshold used to exclude small ROIs, provided as a fraction of the “diameter” parameter	0.5	Advanced parameter. Increase to remove more ROIs, decrease remove fewer ROIs.
Parameter_file	File name used to save the parameter values	Parameters.json	May include a complete path, or only a file name. If only a file name without path is provided, the file will be saved in the directory of the notebook.

### Optional Step 5: Segment ROIs

Once the setup_cell_segmentation notebook has been executed and a parameter file has been generated for ROI segmentation, in order to perform that segmentation on the entire dataset simply open the “notebooks/run_cell_segmentation.ipynb” notebook and direct it to the parameter file created in Step 4. Running the notebook will then segment all of the ROIs as specified in the parameter file and output a new set of tiff files that include an additional layer of ROI masks.

### Step 6: Set-Up Puncta Segmentation

**FIGURE 2:**
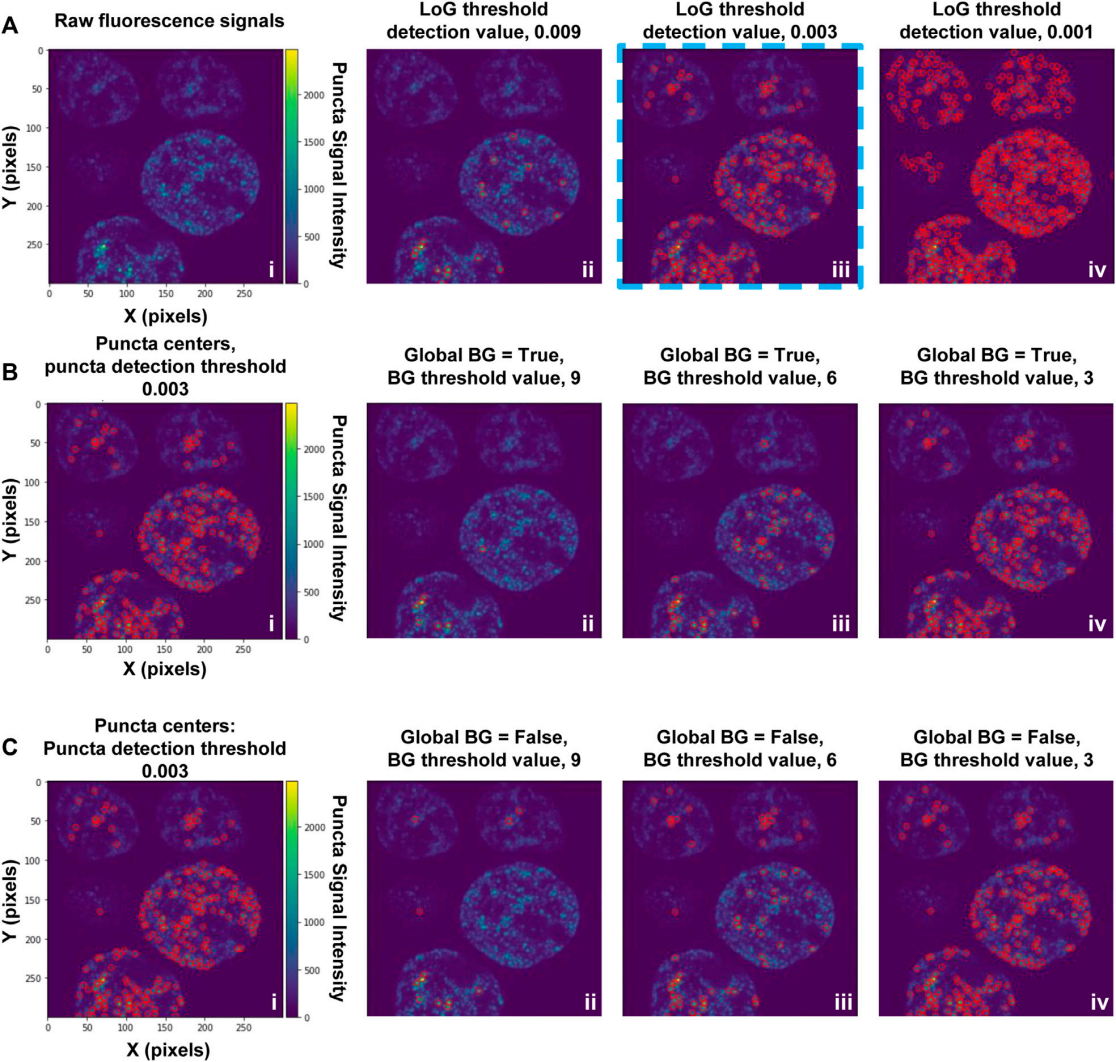
Setting-up puncta centers detection. **(A)** Puncta in fluorescence microscopy images are first identified using a Laplacian of Gaussian (LoG) filter; the detection sensitivity is determined by the LoG “threshold detection” parameter. Puncta centers identified in an image substack for cells expressing G-NHA9 (i) using three settings of the LoG “threshold_detection” parameter are shown (ii–iv). Detection of puncta centers can be further refined through adjustment of background filtering parameters, as illustrated in **(B)** and **(C)**. The examples in **(B)** and **(C)** used a LoG “threshold detection” value of 0.003, as shown in **(A)**, panel iii (blue dashed box). These data are also shown on the left (i) in panels **(B)** and **(C)**. In **(B)** puncta centers were identified using the Global Background (BG) = “True” option, and a range of values of the background (BG) threshold parameter (ii–iv). In **(C)**, puncta centers were identified using the Global Background (BG) = “False” option, and a range of values of the background threshold parameter (ii-iv). With the Global Background (BG) = “True” option **(B)**, a fixed background threshold is applied to all ROI (cell nuclei) in the image, while with the “False” option **(C)**, the background threshold value is calculated for each ROI (cell nucleus) individually.

Puncta segmentation is done by first setting up parameters using the “notebooks/setup_puncta_analysis.ipynb” notebook. This notebook begins with instructions to specify input files. If ROI segmentation was done, specify the parameter file created at the end of Step 4 and the information from that file will be imported into the notebook, including the ROI segmented input images. Otherwise, specify a set of compiled z-stack image files as in Step 3. The notebook will then guide users through selecting a test image, specifying channels of interest for puncta segmentation, and preparing a small image region to test parameters. The first step of puncta segmentation is the detection of puncta centers using a Laplacian of Gaussian (LoG) blob detector. The LoG threshold detection parameter will dictate the sensitivity of the algorithm, with smaller values resulting in more puncta centers being identified, and larger values resulting in fewer puncta centers (Figure 2A). Following identification of puncta centers, the pipeline will refine this selection by comparing their intensities against ROI fluorescent background. The background level is calculated one of two ways: the same value can be used for all cells (Global Background = True, Figure 2B) or each cell can have a unique background intensity level (Global Background = False, Figure 2C).

**FIGURE 3:**
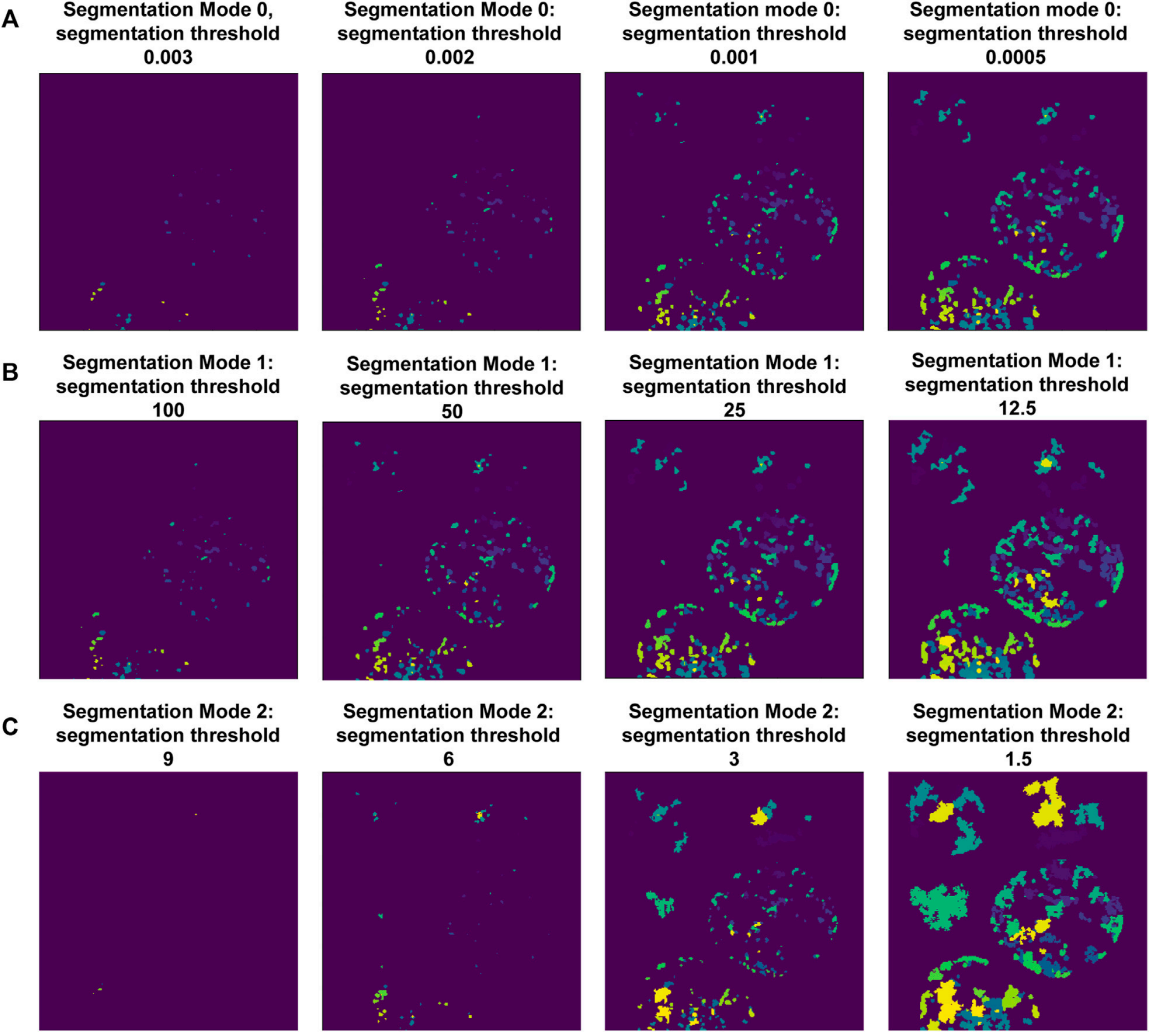
Setting-up puncta segmentation. Puncta Segmentation can be done in three ways, shown using puncta centers identified in Figure 2C, background threshold = 3. **(A)** Mode 0 segments puncta based on the LoG filtered image, using a fixed threshold value. **(B)** Mode 1 segments puncta based on the LoG filtered image using a threshold value relative to the ROI background (LoG) intensity. **(C)** Mode 2 segments puncta based on raw intensity values using a threshold value relative to the ROI background intensity.

Following this, the notebook guides the user through adjusting the puncta segmentation parameters, beginning with the segmentation mode (Figure 3). If no ROIs have been segmented (Steps 4–5), set the segmentation mode to 0; this will analyze all puncta in LoG space using the same parameters according to the user defined segmentation threshold value. If ROIs have been segmented, you may choose mode 1 or 2; this will adjust the segmentation threshold depending on the background fluorescence in the ROIs. Mode 1 will segment puncta based on the LoG transformed image, while Mode 2 will segment puncta based on the original intensity values. This step may result in removal of some additional puncta detected after the previous step. Follow the guidelines in the notebook and Table 2, and inspect the output results to choose an adequate segmentation mode and threshold value for each channel of interest. Following this, additional filters such as the maximum allowed size of puncta and whether puncta outside of ROIs should be removed can be specified. The final parts of this setup protocol are to specify the names of input image channels and the output parameter file (if different from the input parameter file). Execute these parts of the notebook to save the adjusted parameter values, which will be used in the next step.

**TABLE 2:** List of parameters for puncta segmentation and analysis (setup_puncta_analysis.ipynb).

Parameter	Definition	Recommended initial value	Notes
Parameter_file	Parameter file with previously set up ROI segmentation parameters		If ROI segmentation was performed, you can set this to the parameter file name used for ROI segmentation. Alternatively, set this to a new parameter file name and specify the following two parameters: “input_dir” and “cell_segmentation”.
Input_dir	Directory with images to be analyzed		All z-layers and channels for a specific sample must be combined into a single file (see Step 3 of the Protocol Procedure). If ROI segmentation was done, set this to the “output_dir” of the ROI segmentation. Alternatively, ignore this parameter and specify the “parameter_file”.
Roi_segmentation	If True, the last channel of the input images will be used as ROI mask		Set to False if the ROI segmentation step was skipped. Set to True if the images from “input_dir” contain ROI masks as the last channel. Alternatively, ignore this parameter and specify the “parameter_file”.
Output_dir	Output directory to save puncta analysis results		
Puncta_channels	List of channel indices, starting form 0, that will be used to segment puncta		Examples: [1]–puncta will be segmented in the second channel. [2, 3]–puncta will be segmented in the third and fourth channels.
Minsize_um	Minimum target puncta size in µm	0.2	Will be used as the minimum sigma for the Laplacian of Gaussian detector. Decrease to detect smaller puncta, increase to avoid detection of smaller puncta.
Maxsize_um	Maximum target puncta size in µm	2	Will be used as the maximum sigma for the Laplacian of Gaussian detector. Increase to detect larger puncta, decrease to avoid detection of larger puncta.
Num_sigma	Number of sigma values for the Laplacian of Gaussian detection	5	Advanced parameter. Decrease to save computational resources, increase to improve the accuracy of puncta centers detection.
Threshold_detection	Threshold used by LoG detector to exclude low intensity blobs	0.001	Should be close to 0 and can be both positive and negative. Start with threshold_detection = 0 and first adjust minsize_um and maxsize_um to make sure that all puncta of relevant size are detected. After that, gradually increase the value of threshold_detection to remove low-intensity detection. See Figure 3A for examples.
Overlap	Parameter used by the LoG detector to remove the smaller one of two overlapping blobs	1	Advanced parameter. Set to 1 to only remove completely overlapping blobs. Decrease to remove blobs that are further apart. Should be between 0 and 1.
Threshold_background	Threshold used to remove low intensity puncta centers, provided relative to the ROI background value (see “background_percentile”)	3	Example: threshold_background = 3 will remove all puncta centers with fluorescent intensity lower than 3 background values. Set to 0 to keep all puncta centers. Only applied if the ROI masks are provided.
Background_percentile	Intensity percentile (between 0 and 100) used to calculate the background value of the ROI	50	Advanced parameter. 50 corresponds to the median value.
Global_background	If False, the background value is calculated individually for each ROI. If True, the background value is calculated globally as the global_background_percentile of all ROIs	False	Set to False if there is a large range of cell fluorescence values. This will increase sensitivity in cells with low fluorescence and decrease sensitivity in cells with high fluorescence.
Global_background_percentile	Percentile (between 0 and 100) of ROI background values to calculate the global background value	95	Advanced parameter. Only used if global_background = True
Segmentation_mode	Determines the way the “threshold_segmentation” is applied. For mode 0: absolute threshold is applied in LoG space; for mode 1: a threshold relative to the background is applied in LoG space; for mode 2: a threshold relative to the background is applied in image intensity space	0	Advanced parameter. Set to 0 if the background fluorescent signal in all ROIs is relatively uniform. Set to 1 if there is a large range of ROI background fluorescence values.
Threshold_segmentation	Threshold for puncta segmentation. Used in combination with the “segmentation_mode”	0.001 (mode 0)30 (mode 1) 2 (mode 2)	For mode 0, start with values between 0.001 and 0.003; for mode 1, start with values between 20 and 100; for mode 2, start with values between 2 and 3 Decrease or increase to detect more/bigger or fewer/smaller puncta.
Remove_out_of_roi	If True, puncta (parts) that extend beyond ROI will be removed. If False, all puncta will be kept	False	
Maxrad_um	Maximum puncta radius in µm. Used to remove large puncta	None	Set to None to keep all puncta.

### Step 7: Segment and Quantify Puncta

After generating a parameter file using “notebooks/setup_puncta_analysis.ipynb”, segment and quantify the entire dataset using the “notebooks/run_puncta_analysis.ipynb” notebook. To do so, specify in the notebook the path to the parameter file generated at the end of Step 6. Then, execute all the steps in the notebook. It will segment puncta in all the cells in the dataset and generate output files. Output files will include csv files with measurements for individual puncta, and if ROI segmentation was done, additional csv files with measurements on individual ROIs will be generated (Tables 3, 4).

**TABLE 3:** Measurements for individual ROIs (cells or nuclei).

Column name	Definition
Image name	Source file name, including subdirectory
ROI label	Unique ROI (cell or nucleus) ID; matches the pixel value in the ROI segmentation mask
x	the x-coordinate (pixels) of the ROI within the image
y	the y-coordinate (pixels) of the ROI within the image
z	the z-coordinate (pixels) of the ROI within the image
ROI volume pix	Volume (or area, for 2D images) of the ROI in pixels
ROI volume um	Volume of the ROI in µm^3^ (or area in µm^2^, for 2D images)
[Fl] mean intensity per ROI	Average pixel intensity of the [Fl] channel inside the ROI. Calculated for each fluorescent channel in the image
[Fl] integrated intensity per ROI	The sum of all pixel intensities of the [Fl] channel inside the ROI. Calculated for each fluorescent channel in the image
[Fl] mean background intensity	Average pixel intensity of the [Fl] channel in the background (outsize of ROI); will have the same value for all ROI in the image
[Fl] integrated background intensity	The sum of all pixel intensities of the [Fl] channel in the background (outsize of ROI); will have the same value for all ROI in the image
[Fl] entropy	Entropy of the [Fl] channel inside the ROI
Pearson correlation coefficient [Fl] vs. [Fl*]	Pearson correlation coefficient between each pair of fluorescent channels [Fl] and [Fl*] inside the ROI
Pearson correlation *p* value [Fl] vs. [Fl*]	*p*-value for the Pearson correlation coefficient between each pair of fluorescent channels [Fl] and [Fl*] inside the ROI
Mutual information [Fl] vs. [Fl *]	Mutual information between each pair of fluorescent channels [Fl] and [Fl*] inside the ROI
number of [P] puncta	Number of puncta detected in the [P] channel and assigned to the current ROI. This will correspond to the puncta measurements from individual puncta quantification files (Table 4) with matching “Image name” and “cell label” values and “channel” = [P]. Calculated for each channel [P] specified as puncta channel
average [P] puncta volume pix per ROI	Average volume in pixels of puncta detected from channel [P] in the current ROI
average [P] puncta volume um per ROI	Average volume in µm^3^ (or area in µm^2^, for 2D images) of puncta detected from channel [P] in the current ROI
total [P] puncta volume pix per ROI	Total volume in pixels of puncta detected from channel [P] in the current ROI
total [P] puncta volume um per ROI	Total volume in µm^3^ (or area in µm^2^, for 2D images) of puncta detected from channel [P] in the current ROI
average [P] puncta distance to ROI border um per nucleus	Average distance in µm (in 3D) from the puncta’s centers of mass to the ROI border
[Fl] mean intensity inside [P] puncta	The average intensity of the [Fl] channel inside puncta detected in the [P] channel in the current ROI. For [Fl] = [P], this will correspond to the dense phase concentration
[Fl] mean intensity outside [P] puncta	The average intensity of the [Fl] channel outside puncta detected in the [P] channel in the current ROI. For [Fl] = [P], this will correspond to the light phase concentration
[Fl] integrated intensity inside [P] puncta	The sum of all intensities of the [Fl] channel inside puncta detected in the [P] channel in the current ROI.
[FL] integrated intensity outside [P] puncta	The sum of all intensities of the [Fl] channel outside puncta detected in the [P] channel in the current ROI.
Overlap coefficient [P]_[P*]_coloc	Overlap coefficient (overlap over union) for the current ROI for puncta masks detected from each pair of puncta channels [P] and [P*]. This is a measure of colocalization between puncta detected in different channels

**TABLE 4:** Measurements for Individual puncta.

Column name	Defintion
Image name	Source file name, including subdirectory
Puncta label	Unique puncta ID; matches the pixel value in the puncta segmentation mask
ROI label	ID of the ROI (cell or nucleus) the punctum belongs to; 0 corresponds to puncta located outside ROI
Channel	Channel name in which the punctum was segmented
x	the x-coordinate (pixels) of the punctum within the image
y	the y-coordinate (pixels) of the punctum within the image
z	the z-coordinate (pixels) of the punctum within the image
Puncta volume pix	Volume (or area, for 2D images) of the punctum in pixels
Puncta volume um	Volume of the punctum in µm^3^ (or area in µm^2^, for 2D images)
Distance to ROI border um	Distance in µm (in 3D) from the punctum’s center of mass to the border of the cell or nucleus; 0 corresponds to puncta located in the background
[FL] mean intensity per puncta	Average pixel intensity of the [Fl] channel inside the punctum. Calculated for each fluorescent channel in the image
[FL] integrated intensity per puncta	The sum of all pixel intensities of the [Fl] channel inside the punctum. Calculated for each fluorescent channel in the image
Pearson correlation coefficient [Fl] vs. [Fl*]	Pearson correlation coefficient between each pair of fluorescent channels [Fl] and [Fl*] inside the punctum
Pearson correlation *p* value [Fl] vs. [Fl*]	*p*-value for the Pearson correlation coefficient between each pair of fluorescent channels [Fl] and [Fl*] inside the punctum
Mutual information [Fl] vs. [Fl*]	Mutual information between each pair of fluorescent channels [Fl] and [Fl*] inside the punctum. This is a measure of correlation between channels

### Step 8: Verify the Quality of the Cell/Nuclei and Puncta Segmentation

**FIGURE 4:**
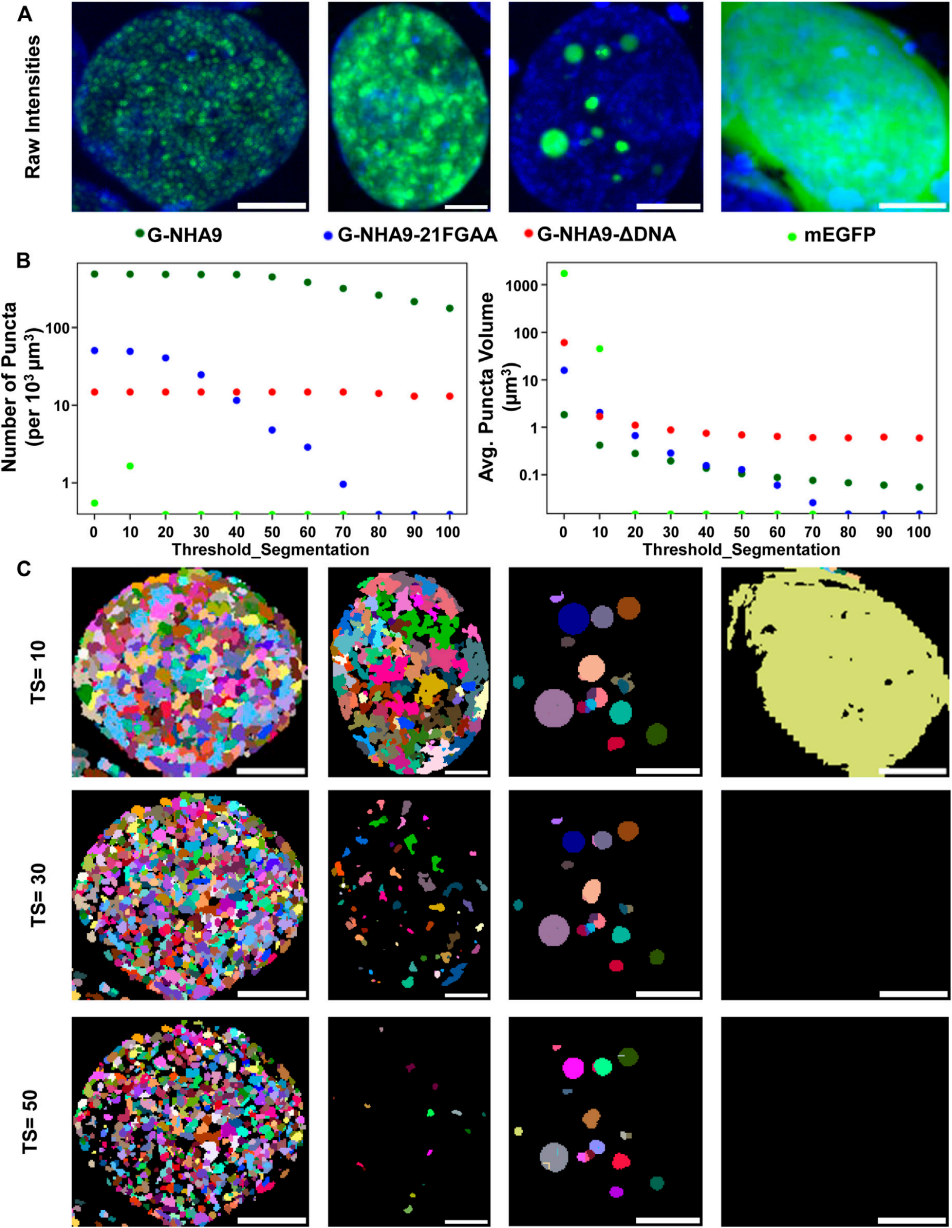
Impact of the “threshold_segmentation” parameter on the number and volume of detected puncta. **(A)** Example cells expressing the G-NHA9 (left), G-NHA9-21FGAA (second from left), and G-NHA9-ΔDNA (second from right) constructs with different sizes of puncta; mEGFP (right) is the negative control. **(B)** Plots showing how extracted features such as average number of puncta per cell (left) and average volume of puncta per cell (right) change with differing segmentation parameters. **(C)** Puncta segmentation masks generated with different threshold_segmentation (TS) values. Scale bars show 5 µm. Puncta segmentation was performed using mode 1, default LoG parameters (num_sigma = 5, minsize_um = 0.2, maxsize_um = 2) and a threshold detection value of 0.002 to identify potential puncta centers. Images are shown as maximum intensity z-projections. Full z-stacks including 3D segmentation can be found in the supplemental files.

The “notebooks/run_puncta_analysis.ipynb” notebook creates tiff files that include the original images along with ROI and puncta segmentation channels. The pixel values in each segmentation mask match the “puncta label” and “ROI label” values from the corresponding csv tables and are useful for identifying specific data points. To examine these masks visually, first open the output tiff files using image viewing software such as FIJI. Then adjust brightness-contrast and/or the lookup tables to improve visibility of each channel. We found that “glasbey” or “glasbey on dark” lookup tables work well for visualizing segmentation masks. Inspect whether the cell/nuclei and puncta masks match expectations. Repeat this inspection for several images from different treatment conditions to ensure consistent performance of the segmentation algorithm and that results align with expectations. It is critical that examined conditions include negative controls (such as diffuse fluorescent signal, and/or samples with no fluorescent signal), to ensure that the parameters are not overly sensitive. If the segmentation results are unsatisfactory, select a small set of representative samples and return to Step 4 and/or Step 6 to adjust the segmentation parameters. Because changes to parameters can alter puncta segmentation, and therefore quantification of puncta features (Figure 4), users are encouraged to implement the same parameter values on the entire dataset across all experimental conditions to maintain quantification accuracy and to ensure that results are comparable. In cases where parameters capable of completely segmenting puncta-containing images while simultaneously not detecting puncta in negative controls cannot be found, we recommend applying the lowest value parameters that return no false puncta in negative controls and acknowledge that this circumstance results in under-segmentation of puncta within some images. While inspecting results, note the image name and cell ID of any dying or obviously aberrant cells. A list of these cells can be supplied to a post-processing script in Step 10 to exclude them from final analysis.

### Optional Step 9: Calibration of Fluorescence Intensity Versus Concentration

For biologically relevant studies, it may be necessary to estimate concentrations of fluorescently-labeled biomolecules in cells treated under different conditions. To estimate concentrations of fluorescently-labeled protein inside and outside of puncta, a conversion factor that establishes the relationship between fluorescence intensity and fluorescent protein (e.g., mEGFP) concentration needs to be calculated. Prepare pure solutions of known concentrations of fluorescent protein in imaging media and collect images using parameters that are identical to those used in the experimental data collection (laser power wattage, acquisition time, etc.). When preparing these samples, it is important to account for factors that influence fluorescence intensity such as crowding, pH, and concentration; these factors can influence the intrinsic fluorescence of the fluorescent-label in the cellular setting (Kneen et al., 1998; Leiderman et al., 2006; Morikawa et al., 2016). We provide an example notebook, “notebooks/calibrate_intensities.ipynb” to match fluorescent intensities to protein concentrations (e.g., for the mEGFP channel). With this, a standard curve of the relationship between apparent fluorescence intensity and fluorescent protein concentration can be established, which can then be used to calculate a conversion factor. This parameter can be used to determine the apparent concentrations of labeled biomolecule inside (dense phase) and outside (light phase) of puncta when the relationship between fluorescence intensity and concentration is linear. Note that the exact concentration is not necessary to determine thermodynamic parameters such as partition coefficients.

### Optional Step 10: Calculation of Thermodynamic Characteristics

This processing step enables pipeline users to generate multiple plots in several formats that allow comparison between experimental conditions. Immediate comparisons that can be made by this step are comparisons of basic parameters of cells such as: number of puncta, average volume of puncta, and average fluorescence intensity of a given fluorophore (FL) within cells. To facilitate further comparisons, data can be analyzed to derive the partition coefficient (K_p_) for the fluorescently-labeled biomolecule within puncta Eq. 1 and partition coefficients are then used to derive the Gibbs free energy of transfer into puncta (ΔG_Tr_) Eq. 2, where R (1.9872) is the gas constant in units of kcal/mol and T is temperature in Kelvin units.

$$\;K_{p}=\frac{\left[\mathrm{DP}\right]}{\left[\mathrm{LP}\right]}=\;\frac{\left[\mathrm{FL}\right]\;\mathrm{mean}\;\mathrm{intensity}\;\mathrm{inside}\left[\mathrm{FL}\right]\;\mathrm{puncta}}{\left[\mathrm{FL}\right]\;\mathrm{mean}\;\mathrm{intensity}\;\mathrm{outside}\left[\mathrm{FL}\right]\;\mathrm{puncta}}$$ (1)

$$\;\Delta G_{\mathrm{Tr}}=-\mathrm{RTln}\left(K_{p}\right)$$ (2)

To compile output data, calculate thermodynamic parameters, and compare datasets, we developed R functions that read output files from previous steps and performs these calculations. This function can be found in the “scripts/thermodynamic_characterization/puncta_thermo_calc.Rmd” R notebook provided. This script also generates plots to compare datasets. To run this analysis, first load the requisite packages and the function in R. Then call the function and provide the necessary inputs: cell. data = “PATH to cell data csv file”, puncta. data = “PATH to puncta data csv file”, cell. channel = “Name of ROI signal”, puncta. channel = “Name of puncta signal”, factor_conc = [[Conversion factor from intensity to µM]] (optional), and temp = [[temperature in Kelvin units]]. Assign output of the function to a dataframe, which can then be exported to a spreadsheet.

### Step 11: Statistical Analysis and Comparisons

To determine the statistical significance of differences between the features of puncta imaged in different biological replicates, or under different biological conditions, use the provided R package “scripts/statistical_analysis/library.R”. An example of how to prepare and run the package is also provided as “scripts/statistical_analysis/runPuncta.R”. To utilize the package, first read the cell-level data for multiple biological conditions into a data frame in R. Then call the function and specify the following inputs: the column names of the characteristics to be compared (y.var), the condition variable (cond.var), the image subsample/position (subsamp.var), a vector with names of the conditions to include in the analysis (cond.inc.), the name of the transformation (trans.name, examples include “identity” or “log”), and an indicator of whether the y-variable is a count variable (y.count, T or F). Based on these inputs, compare. puncta.R will determine the proper statistical tests to perform and compute statistical estimates of the effect of the biological conditions on y with confidence intervals and *p*-values. It will then return the input data, statistical results, and a brief narrative describing the results.

## Results

### Software Design

We provide the full image analysis pipeline as an open-source Python package “Punctatools” (https://github.com/stjude/punctatools). It is solely dependent on open-source tools, such as Cellpose (Stringer et al., 2021), scikit-image (van der Walt et al., 2014), and intake_io (Höckendorf and Medyukhina, 2022), all of which will be installed automatically at setup. The pipeline consists of tools that perform several major functions (Figure 1): data staging, segmentation of the regions of interest (ROIs; e.g., individual cells or nuclei), puncta segmentation, and puncta quantification (discussed below).

ROI segmentation is done using Cellpose (Stringer et al., 2021), a deep-learning-based algorithm for cell and nucleus segmentation. This step is applied to segment either cells or nuclei, depending on the fluorescent signal used (e.g., Hoechst for segmenting nuclei in the examples provided here). Following segmentation of ROIs in individual *z*-layer images, they are combined in three dimensions (when the two-dimensional segmentation mode was used). Optionally, tools are provided to exclude small ROIs (e.g., those corresponding to aberrant or dying cells) and/or ROIs near the edges of images in the x- and *y*-axis dimensions (see Supplementary Meterial S1).

Segmentation of puncta within ROIs is done through the following steps (Figures 2,3; Supplementary Meterial S1): 1) a Laplacian of Gaussian (LoG) filter is applied to identify candidate puncta centers (Figure 2A); 2) intensity-dependent puncta center filtering is performed based on contrast to the background signal of the ROI (Figures 2B,C); 3) thresholding of the image, followed by a seeded watershed segmentation is applied to the LoG transformed (Figures 3A,B) or raw fluorescence intensity signal (Figure 3C); and 4) optional filtering of puncta is done based on their size or extension beyond the ROI mask (see Supplementary Meterial S1).

After ROI and puncta segmentation, the Punctatools pipeline performs quantitative measurements on ROIs (Table 3) and puncta (Table 4). For each punctum, Punctatools quantifies location, volume, distance to ROI border, as well as the mean and integrated fluorescence intensities of all pixels in all detection channels within the segmented punctum. The values of these parameters are summarized for individual ROI. Punctatools also quantifies the mean and integrated fluorescence intensities for all pixels within each ROI, as well as all pixels inside (dense phase) and outside (light phase) of puncta for each ROI; if a calibration curve was established relating fluorescence intensities with molar concentrations (e.g., for the mEGFP channel; see Step 9), these values can be converted to molar concentration units. In our example data, ROIs of interest are cell nuclei and the biomolecules of interest (G-NHA9 constructs) are fluorescently-labeled with mEGFP. Therefore, the parameters described above give the apparent average total nuclear mEGFP concentration for all nuclei in the images being analyzed, and corresponding dense and light phase concentrations. Furthermore, the pipeline will report correlation statistics (e.g., Pearson correlation coefficients, mutual information) for each pair of channels. This calculation is performed and reported for both ROIs and puncta. Additionally, if multiple puncta channels are segmented, the pipeline will calculate the overlap coefficient (overlap over union) between segmented puncta for each ROI.

The parameters for all steps of ROI and puncta segmentation are interactively adjusted using the provided setup notebooks and allow users to tune pipeline performance until results agree with their expert assessment (see Tables 1, 2 for the full list of adjustable parameters). The sensitivity, computational expense, and limits of detection will be determined by these user-defined parameters. For example, the parameters that determine the LoG transformation (num_sigma, overlap, threshold_detection, maxsize_um, and minsize_um) allow users to adjust the pipeline to detect puncta within the expected size range. Because changes in the analysis parameters result in differences in quantifications of puncta features (Figure 4), when comparing the puncta features of different conditions, all conditions need to be analyzed with the same parameters.

In addition to the Punctatools package, we provide the set of jupyter notebooks (Python language scripts), which we used for the analyses shown in this manuscript. The notebooks include step-by-step guides on how to adjust ROI and puncta segmentation parameters to adapt the workflow for particular experimental conditions (e.g., cell type, type of fluorescently-labeled biomolecule, location of puncta formed by the labeled biomolecule with cells, etc.).

We also developed an R-function to extract thermodynamic parameters from the raw pipeline output (puncta_thermo_calc.Rmd). The function first reads the ROI and puncta datasets generated in previous steps for a given experimental condition, along with parameters needed to calculate the relevant characteristics of each cell. These include the apparent concentration of the fluorescently-labeled biomolecule (e.g., a mEGFP-labeled protein) in the ROI and the corresponding dense and light phase, the partition coefficient (K_p_), and the Gibbs free energy of transfer (ΔG_Tr_). Data generated by this function can be exported to a spreadsheet for examination, or directly plotted in R to make comparisons between conditions.

Finally, we developed an R package to assess the statistical significance of differences in values of puncta parameters generated using the procedures discussed above for single replicates of images recorded under different biological conditions. The package can be found in the Punctatools github repository “scripts/statistical_analysis/library.R”. The package defines a function image.stats that compares ROI-level imaging characteristics across biological conditions. The input data is first passed as the dset argument to compare. puncta. The function then transforms the data as specified (trans.name) and subdivides it to include the biological conditions of interest (cond.var). Next, it fits a linear mixed effects model if y is not an integer variable (using lmer of the lme4 package) or a general linear mixed effects model with a Poisson distribution and log-link if y is a count variable (using glmer of the lme4 package). In each case (when y is an integer variable or not), the model represents the substantial variability between technical replicates in y (subsamp.var) as a random effect and the biological comparison across conditions of primary scientific interest (cond.var) as a fixed effect. The package then computes statistical estimates of the effect of the biological conditions on y with confidence intervals and *p*-values. Finally, the package returns these statistical results, copies of the input data and specifications, and a short narrative describing the results of the statistical analysis. In future work, we intend to extend this function to appropriately manage data from experiments with multiple biological replicates per condition. As the data collection for these studies becomes streamlined and less cumbersome, we encourage investigators to include multiple biological replicates in their studies to enhance statistical reliability and scientific rigor.

### Example Results

**FIGURE 5:**
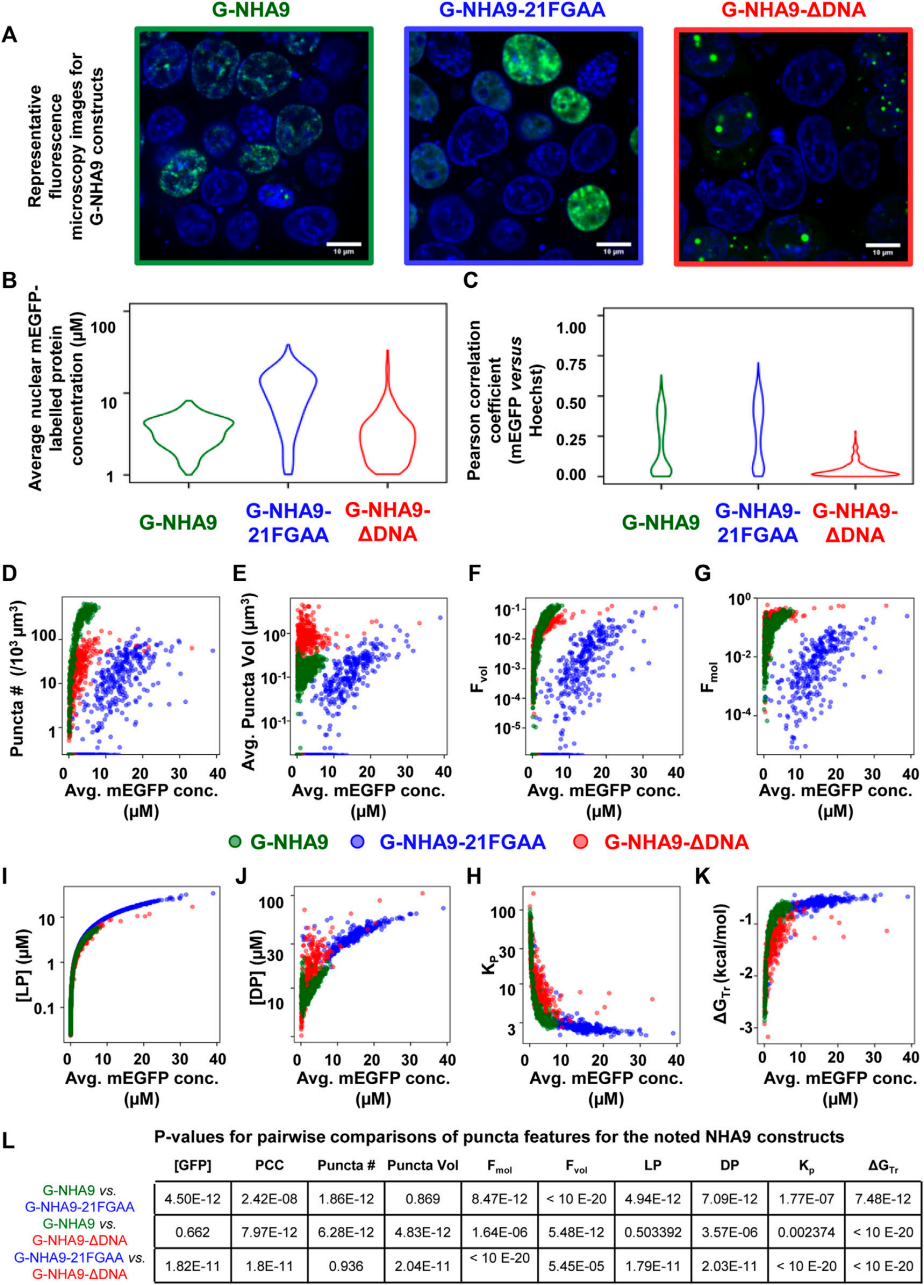
Example Punctatools pipeline output. **(A)** Exemplary microscopy images illustrating differences in puncta features for G-NHA9 (green box, *n* = 1,004), G-NHA9-21FGAA (blue box, *n* = 880), and G-NHA9-ΔDNA (red box, *n* = 849). The fluorescence signals for the G-NHA9 constructs are illustrated in green and those for Hoechst dye are shown in blue. The Punctatools pipeline quantifies raw images to extract parameters such as, **(B)**, the apparent average nuclear concentration per cell of the fluorescently-labeled biomolecule (mEGFP) and **(C)**, Pearson correlation coefficients (PCC) between fluorescent channels (the Hoechst dye and mEGFP channels in these examples). Additional functions within Punctatools generate plots reporting features such as, **(D)** Puncta number; **(E)** Puncta volume; **(F)** Puncta volume fraction (F_vol_); **(G)** Puncta mole fraction (F_mol_); **(H)** Light phase concentration (LP); **(I)** Dense phase concentration; **(J)** Partition coefficient (K_p_); or **(K)**, ΔG_Tr_ versus the apparent average concentration of the fluorescently-labelled biomolecule (mEGFP in these examples) per ROI (cell nucleus in these examples). **(L)** The pipeline also determines the statistical significance of differences between these values for different conditions (three G-NHA9 constructs in these examples), including *p*-values. Average mEGFP concentrations shown on the graph *x*-axes are estimates derived from calibration, and are subject to the limitations of this calibration as noted in the main text.

Successful implementation of this pipeline up to Step 8 will return multiple results files. Amongst these are annotated image-format files that depict the masks generated for ROIs and puncta, in addition to the original intensity information; these files are stored within the puncta subdirectory. Masks are helpful for visually inspecting the results to determine whether ROIs (e.g., nuclei in our G-NHA9 construct examples) were properly segmented, and that puncta were correctly identified and segmented. Additionally, there are numerous spreadsheets that report the extracted puncta features for the different conditions being tested. One set of spreadsheets details parameters for individual puncta, noting volume (in pixels^3^ and μm^3^), x-, y-, *z*-axis position, average and integrated fluorescence intensity, etc. Another set of files reports information relevant to the behavior of condensates formed in cells by the fluorescently-labeled biomolecule(s). For example, average values for puncta features within each ROI, average fluorescence intensity in the ROI, and correlation between fluorescence intensities of the different channels within the ROI. Further processing (as in Steps 9–11) converts this information into features relevant for thermodynamic analysis. We illustrate some of these results for images of live HEK293T cells expressing three different G-NHA9 constructs (Figure 5A) analyzed using the Punctatools pipeline. For example, as discussed under Procedure (Step 9), if a standard curve for the relationship between fluorescence intensity of the fluorescent label (mEGFP in our case) and molar concentration is available, the average fluorescence intensity per ROI (nuclei in our case) can be converted to average molar concentration within each ROI. The results show that the average nuclear concentration of the mEGFP-labeled constructs for individual cells varies from 1 μM to over 10 μM and that the concentration ranges and distributions differ between the constructs (Figure 5B). Also, the extent of positional overlap of the fluorescence signals of the mEGFP-labelled G-NHA9 constructs and the Hoechst dye, which stains chromosomal DNA, as given by the Pearson correlation coefficient, differs for the three G-NHA9 constructs (Figure 5C). The conversion of fluorescence intensity to molar concentration enables examination of the dependence of puncta features on the concentration of the puncta-forming biomolecule—on a per ROI basis (per cell nucleus in our examples). We illustrate this feature of Punctatools on mEGFP concentration-dependence of puncta number, puncta mole fraction (e.g., the fraction of mEGFP-labeled biomolecules within puncta) and ΔG_Tr_ (e.g., the thermodynamic favorability of the labeled biomolecule partitioning into puncta) for the three G-NHA9 constructs (Figures 5D–F). Finally, we determine statistical significance of the differences between puncta features using the statistics tools within the Punctatools pipeline (Figure 5G). In summary, the functionalities included in Punctatools enable evaluation and optimization of image analysis performance and, assuming suitable performance is achieved, detailed analysis of features of puncta formed by fluorescently-labeled biomolecules in live cells.

### Comparison to Other Methods

FIJI is an open-source platform used to view and analyze image data with numerous plugins that provide additional functionality. We used the “Coloc 2” plugin to confirm the Pearson correlation coefficient (PCC) values output by our pipeline. To do so, we first imported the Cellpose ROIs into FIJI, then isolated the regions from the surrounding data and ran the plugin. We compared the PCC values generated by “Coloc 2” with those generated by our pipeline for each ROI in a sample image and found them to be identical, confirming robustness of our pipeline’s calculations. Additionally, we compared the results of the Punctatools pipeline’s segmentation against manual segmentation of puncta done by drawing ROIs in FIJI, and quantified each cell as defined by Cellpose. We found the results to be in good agreement (Supplementary Figures 1B,C).

**FIGURE 6:**
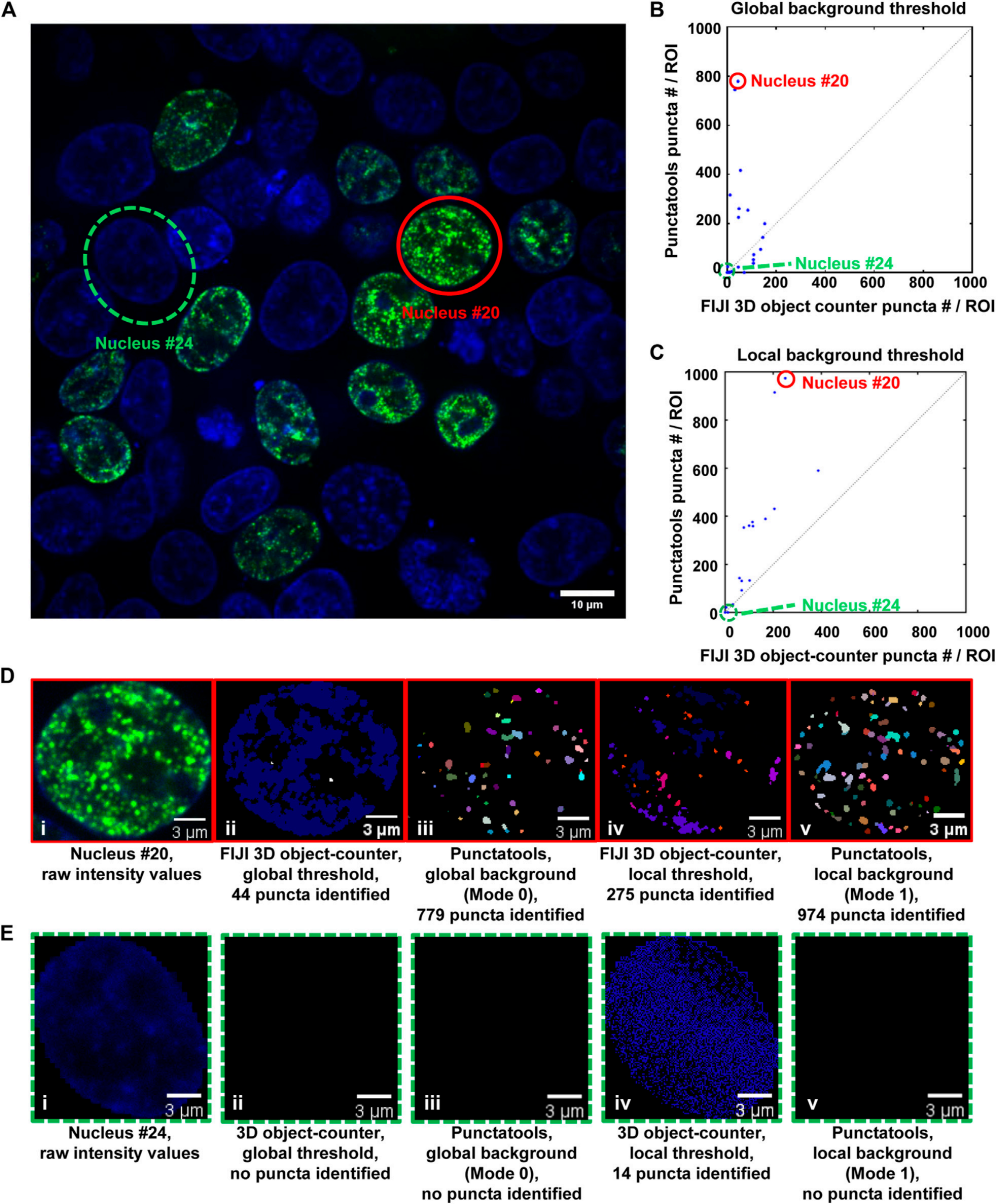
Comparison of puncta segmentation performance: Punctatools versus FIJI 3D object-counter. **(A)** A single z-layer image of puncta in cells expressing G-NHA9 (the G-NHA9 fluorescence signals are illustrated in green and those for Hoechst dye are shown in blue); two ROIs (Cells #20 (solid red circle) and #24 (dashed green circle)) in this image were used for the performance comparisons presented in other panels. Cells #20 and #24 display many puncta and no puncta, respectively. Analysis of the 3D z-stack data to quantify puncta features for individual ROIs (57 nuclei total) resulted in different numbers of puncta by the Punctatools pipeline and FIJI 3D Object counter differ; in **(B)**, a constant, global background threshold was used by Punctatools (Mode 0) and the FIJI 3D object-counter and in **(C)**, the background thresholds were determined by the two algorithms for each ROI (cell nucleus; Mode 1 for Punctatools). In **(B)** and **(C)**, the number of puncta identified by Punctatools for each ROI is plotted on the *x*-axis and that determined by the FIJI 3D object-counter on the *y*-axis. **(D)** Raw image data for Cell #20 displaying many puncta (a single z-slice is shown, (i) and puncta segmentation masks generated using the FIJI 3D object counter (ii, iv) or Punctatools (iii,v) using global (ii,iii) or local, ROI-specific (iv,v) background thresholds. **(E)** as in **(D)** for Cell #24, which did not express G-NHA9 and does not display puncta. In **(D)** and **(E)**, the G-NHA9 fluorescence signals are illustrated in green and those for Hoechst dye are shown in blue. The masks for segmented puncta in **(D)** are illustrated in multiple colors.

We also used a FIJI plugin to compare our pipeline’s puncta segmentation against conventional thresholding approaches. “3D object counter” identifies and counts 3D objects in a set of data by first suggesting a recommended threshold value and then separating “objects” from the background based on the distribution of intensity values in the analyzed region. To segment puncta using this plugin, we first isolated individual cell nuclei from an acquired z-stack of puncta-displaying cell nuclei using the regions generated from Cellpose as generated in Step 5. We then analyzed each cell nucleus in this file with 3D object counter in two different ways: with a threshold automatically determined from the collective intensity distribution in the entire image-stack (Global Sensitivity), and with a threshold automatically determined from each individual cell nucleus’s intensity distribution (Local Sensitivity). We then compared the results between these analyses and the analyses of two different configurations of our pipeline: one that analyzes cell nuclei in the context of the entire file (Mode 0, Global Background = True, Figure 6B) and a configuration that analyzes cell nuclei individually (Mode 1, Global Background = False, Figure 6C). In cell nuclei that display obvious puncta, such as nucleus #20 (Figure 6D), the Punctatools pipeline found more puncta (974 and 779, with local and global background options, respectively) than the FIJI 3D Object Counter method (44 and 275, respectively). In contrast, in a low expressing cell with no apparent nuclear puncta (nucleus #24), the Punctatools pipeline found no puncta while the 3D Object counter method found 14 puncta when evaluating the cell nucleus with a local threshold (Figure 6E). In addition, we found that for both thresholding methods, the Punctatools pipeline returned segmentation masks that were truer representations of puncta shape, and the pipeline performance was superior in segmenting separate puncta in areas where multiple puncta existed close to one another. In conclusion, we determined that the LoG-based method of determining puncta segmentation used in Punctatools is superior to conventional intensity-thresholding methods.

Puncta analysis could potentially be simplified by 2D projection from 3D data prior to analysis. However, 2D projection produced inaccuracies in quantification of key quantities such as volume fraction and partition coefficient (Supplementary Figures 1D,E).

There are multiple existing software packages designed to facilitate algorithmic analysis of fluorescence microscopy data. For example, there are numerous open-source plugins for FIJI that recapitulate many of the analysis steps performed in the Punctatools pipeline. Additionally, CellProfiler (Stirling et al., 2021) contains a suite of image analysis tools designed to process and analyze large batches of image data. These open-source tools have been used to develop many different protocols that perform a large variety of analytical tasks on cell images and can execute these protocols on large sets of data and could be used to create pipelines comparable to the Punctatools pipeline.

## Discussion

Our image analysis pipeline was developed to facilitate objective, automated analysis of fluorescence microscopy images of biomolecular condensates formed through the process of LLPS. We tailored the performance of our pipeline to accommodate the physical features of biomolecular condensates; for example, the identified condensates, or puncta, are analyzed over their entire three-dimensional volumes and their boundaries are identified using a Laplacian of Gaussian filter applied to their fluorescently-labeled components. Quantification of entire puncta volumes enables analysis of the total puncta volume relative to the remaining nuclear volume, which is termed the puncta volume fraction (F_vol_). Our pipeline also quantifies the intensity of the signal for the fluorescently-labeled biomolecule within puncta, termed the dense phase, and within the surrounding ROI (the nucleoplasm in our examples), termed the light phase. These fluorescence intensities can be converted to apparent molar concentrations in the respective phases if an appropriate standard curve is available (e.g., recorded using purified mEGFP for our examples). The availability of these concentrations enables the mole fraction of the fluorescently-labeled biomolecule within puncta (F_mol_) to be determined (Figure 5E). These parameters, F_vol_ and F_mol_, are not available from other image analysis software packages. Another advantage of our image analysis pipeline is that it is more sensitive to the presence of puncta than other, intensity-base detection methods (Figure 6). Our pipeline detects puncta by using the Laplacian of Gaussian transformation, which enables detection of sharp fluorescence intensity fluctuations that distinguish the puncta dense phases from the surrounding light phase. In the simplest case, the theory of liquid-liquid phase separation provides that the concentration of the fluorescently-labeled biomolecule that forms puncta has a uniform concentration in the light phase and a uniform but higher concentration in the dense phase. The ratio of dense to light phase concentrations is the partition coefficient (K_p_) and its value in condensates can range from a few-fold to over 100-fold. Our pipeline detected hundreds of puncta formed in cells by the NHA9 fusion oncoprotein while intensity-based image analysis methods detected significantly fewer puncta (Figure 6). While we illustrate the performance of our image analysis pipeline using exclusively nuclear puncta-forming proteins, it is adaptable to identifying and analyzing condensates formed outside of the nucleus with appropriate experimental design (e.g., by providing a fluorescent signal for segmentation of the entire cell volume). Therefore, we believe that the Punctatools image analysis pipeline discussed herein will be useful in the analysis of cellular condensates formed by many other fluorescently-labeled biomolecules.

The quantitative information from Punctatools pipeline provides detailed insight into the phase separation behavior of biomolecules in cells. For example, we enumerate the average fluorescence intensity per unit volume for fluorescently-labeled biomolecules which, when a standard curve relating fluorescence intensities to molar concentrations is available, allows the concentration dependence of puncta features to be examined. Puncta number (Figure 5D) increases for the three G-NHA9 constructs (G-NHA9, G-NHA9-21FGAA and G-NHA9-ΔDNA) with increasing nuclear expression level, but these dependencies vary. The G-NHA9-ΔDNA construct, which interacts with other proteins in the nucleus but does not bind DNA, forms fewer puncta that are larger (Figure 5E) than those formed by G-NHA9. In contrast, the G-NHA9-21FGAA construct, which harbors 21 pairs of mutations in the FG motifs that drive phase separation, forms fewer puncta than G-NHA9 and only at significantly higher protein expression levels. These results, based upon quantitative image analysis using the Punctatools pipeline, illustrate how altering the functional properties of the NHA9 fusion oncoprotein through mutation (e.g., by altering interactions with DNA or proteins, respectively, with G-NHA9-ΔDNA and G-NHA9-21FGAA) affects LLPS and puncta features in cells. Quantitative analysis of puncta within cell images also provides access to information on the volume occupied by puncta within the ROI (nuclei in our studies). For example, puncta formed by the G-NHA9 constructs occupy only a very small fraction of the nuclear volume (Figure 5F), especially at low expression levels, although this fraction increases to almost 0.1 at the highest expression levels. However, the fraction of mEGFP-labeled protein molecules within puncta (Figure 5G) exceeds 0.1 at the higher expression levels due to preferential partitioning within the phase-separated condensates, as given by the partition coefficient (K_p_, Figure 5H). We note, however, that G-NHA9 construct partitioning within puncta declines sharply with increasing expression level (Figure 5H) because the mEGFP-labeled protein concentration increases more steeply in the light phase ([LP], surrounding puncta) than in the dense phase ([DP], within puncta (Figures 5I,J). This is due to declining thermodynamic favorability of incorporation of mEGFP-labeled G-NHA9 molecules within puncta with increasing expression level (e.g., ΔG_Tr_ values become less negative, Figure 5K), which is a hallmark of multicomponent phase separation driven by heterotypic interactions (Riback et al., 2020). Thus, the quantitative analyses enabled by Punctatools provides detailed insight into relationships between LLPS mechanisms and the physical and thermodynamic features of cellular puncta.

It is now recognized that large regions of the interior of cells are occupied by biomolecular condensates that form through LLPS and perform diverse biological functions. While the basic principles underlying LLPS by biomolecules are understood, tools to quantitatively compare the features of the different types of condensates that form through LLPS are limited. Fluorescence microscopy is commonly applied in studies of condensates formed in cells by fluorescently-labeled biomolecules but often only qualitative analyses of the resulting images are performed. While many types of image analysis software tools are available for quantitative analyses of punctate structures in cells, we are not aware of any that have been developed with the principles of LLPS in mind. The Punctatools image analysis pipeline presented here was developed to enable the identification and segmentation of cellular puncta formed through LLPS, which in turn provides access to the wealth of quantitative physical and thermodynamic features discussed above. While the example data and analyses we present were generated through studies of fluorescently-labeled proteins that form nuclear puncta, our pipeline is adaptable for studies of condensates that form within the cytoplasm with appropriate experimental design. Therefore, we propose that Punctatools will be useful in studies of the growing numbers of biomolecules that perform their function(s) in the setting of phase separated condensates.

## Data Availability

The original contributions presented in the study are included in the article/Supplementary Material, further inquiries can be directed to the corresponding authors.
