# Supplementary figures and images for: An Image Analysis Pipeline for Quantifying the Features of Fluorescently-Labeled Biomolecular Condensates in Cells
Source: Front Bioinform. 2022 Jun 6;2:897238. doi: 10.3389/fbinf.2022.897238 (PMC9580871; doi:10.3389/fbinf.2022.897238)

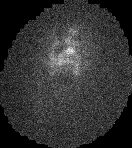

Supplement: Supplementary file 1 [file DataSheet3.ZIP › G-NHA9-DDNA_Fig4_Zstacks/AAG_TS_0.tif]

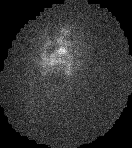

Supplement: Supplementary file 1 [file DataSheet3.ZIP › G-NHA9-DDNA_Fig4_Zstacks/AAG_TS_10.tif]

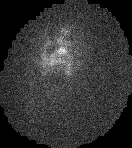

Supplement: Supplementary file 1 [file DataSheet3.ZIP › G-NHA9-DDNA_Fig4_Zstacks/AAG_TS_100.tif]

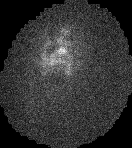

Supplement: Supplementary file 1 [file DataSheet3.ZIP › G-NHA9-DDNA_Fig4_Zstacks/AAG_TS_20.tif]

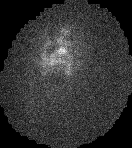

Supplement: Supplementary file 1 [file DataSheet3.ZIP › G-NHA9-DDNA_Fig4_Zstacks/AAG_TS_30.tif]

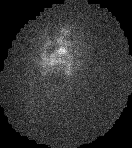

Supplement: Supplementary file 1 [file DataSheet3.ZIP › G-NHA9-DDNA_Fig4_Zstacks/AAG_TS_40.tif]

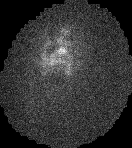

Supplement: Supplementary file 1 [file DataSheet3.ZIP › G-NHA9-DDNA_Fig4_Zstacks/AAG_TS_50.tif]

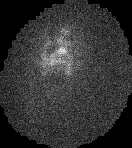

Supplement: Supplementary file 1 [file DataSheet3.ZIP › G-NHA9-DDNA_Fig4_Zstacks/AAG_TS_60.tif]

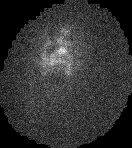

Supplement: Supplementary file 1 [file DataSheet3.ZIP › G-NHA9-DDNA_Fig4_Zstacks/AAG_TS_70.tif]

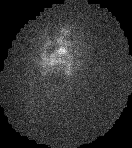

Supplement: Supplementary file 1 [file DataSheet3.ZIP › G-NHA9-DDNA_Fig4_Zstacks/AAG_TS_80.tif]

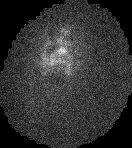

Supplement: Supplementary file 1 [file DataSheet3.ZIP › G-NHA9-DDNA_Fig4_Zstacks/AAG_TS_90.tif]

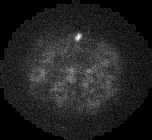

Supplement: Supplementary file 2 [file DataSheet4.ZIP › G-NHA9_WT_Fig4_Zstacks/NHA9_WT_TS_0.tif]

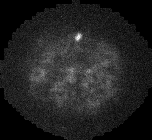

Supplement: Supplementary file 2 [file DataSheet4.ZIP › G-NHA9_WT_Fig4_Zstacks/NHA9_WT_TS_10.tif]

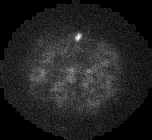

Supplement: Supplementary file 2 [file DataSheet4.ZIP › G-NHA9_WT_Fig4_Zstacks/NHA9_WT_TS_100.tif]

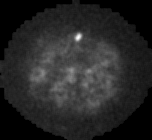

Supplement: Supplementary file 2 [file DataSheet4.ZIP › G-NHA9_WT_Fig4_Zstacks/NHA9_WT_TS_20.tif]

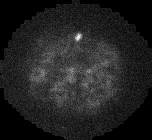

Supplement: Supplementary file 2 [file DataSheet4.ZIP › G-NHA9_WT_Fig4_Zstacks/NHA9_WT_TS_30.tif]

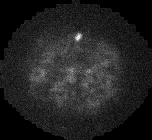

Supplement: Supplementary file 2 [file DataSheet4.ZIP › G-NHA9_WT_Fig4_Zstacks/NHA9_WT_TS_40.tif]

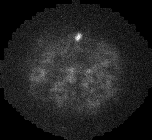

Supplement: Supplementary file 2 [file DataSheet4.ZIP › G-NHA9_WT_Fig4_Zstacks/NHA9_WT_TS_50.tif]

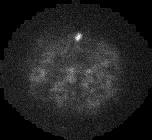

Supplement: Supplementary file 2 [file DataSheet4.ZIP › G-NHA9_WT_Fig4_Zstacks/NHA9_WT_TS_60.tif]

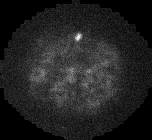

Supplement: Supplementary file 2 [file DataSheet4.ZIP › G-NHA9_WT_Fig4_Zstacks/NHA9_WT_TS_70.tif]

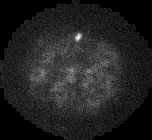

Supplement: Supplementary file 2 [file DataSheet4.ZIP › G-NHA9_WT_Fig4_Zstacks/NHA9_WT_TS_80.tif]

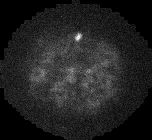

Supplement: Supplementary file 2 [file DataSheet4.ZIP › G-NHA9_WT_Fig4_Zstacks/NHA9_WT_TS_90.tif]

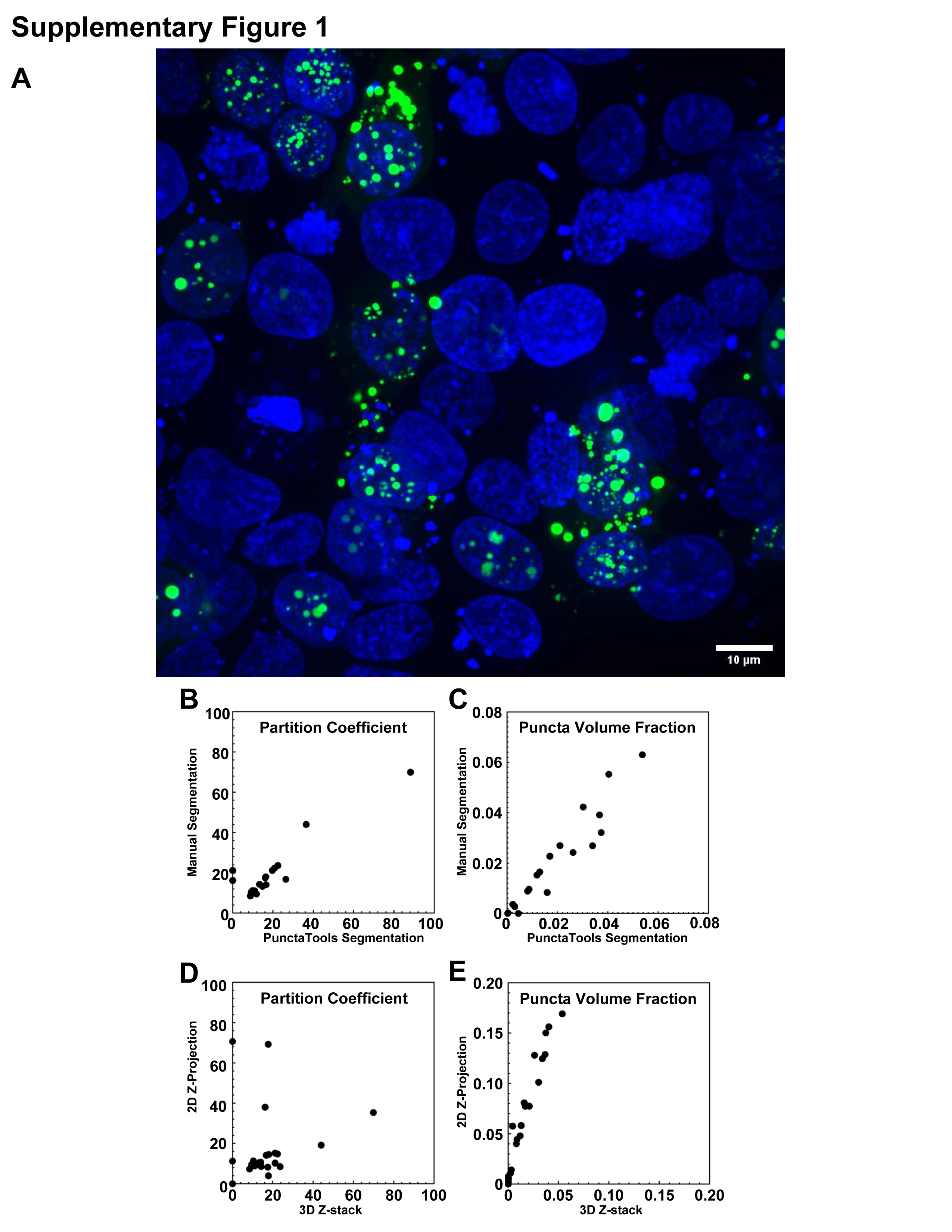

Supplement: Supplementary file 3 [file Image1.TIF]

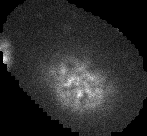

Supplement: Supplementary file 5 [file DataSheet5.ZIP › mEGFP_Fig4_Zstacks/GFPempty_TS_0.tif]

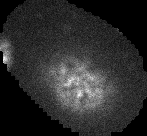

Supplement: Supplementary file 5 [file DataSheet5.ZIP › mEGFP_Fig4_Zstacks/GFPempty_TS_10.tif]

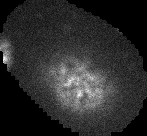

Supplement: Supplementary file 5 [file DataSheet5.ZIP › mEGFP_Fig4_Zstacks/GFPempty_TS_100.tif]

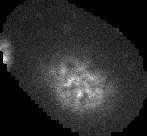

Supplement: Supplementary file 5 [file DataSheet5.ZIP › mEGFP_Fig4_Zstacks/GFPempty_TS_40.tif]
