# Supplementary material for: An Image Analysis Pipeline for Quantifying the Features of Fluorescently-Labeled Biomolecular Condensates in Cells
Source: Front Bioinform. 2022 Jun 6;2:897238. doi: 10.3389/fbinf.2022.897238 (PMC9580871; doi:10.3389/fbinf.2022.897238)
Supplement: Supplementary file 6 [file DataSheet1.DOCX]

Supplementary Methods

# ROI segmentation:

ROI segmentation is done using cellpose and is done either in a 3D mode or 2D mode, depending on the user choice. If the 3D mode is chosen, cellpose outputs 3D labeled masks of cells or nuclei. If the 2D mode is chosen, cellpose segments and labels cells / nuclei in individual z-layers. In this case, the labels for the same cell in different z-layers do not match and need to be relabeled to generate 3D labeled masks, which is done as follows:

1. Calculate the total foreground (cell) area in each z-layer.

2. Detect the layer with the largest total cell area and extract the cellpose labels from this layer; if the z-stack has more than 21 layers, the first and the last 10 layers are not considered in this step.

3. Convert all z-layer labels to binary masks (0 for the background, 1 for cells).

4. Multiply each binary mask by the labels from step 2.

This relabeling procedure works well for samples where cells/nuclei are arranged in one layer and don’t lie on top of each other. This procedure combined with the 2D mode of cellpose is more memory efficient and should be preferred for such “thin” samples. The 3D mode of cellpose was more accurate in some of our tests, but it required more GPU memory, or significantly more computational time if the available GPU memory was not sufficient and we had to use the CPU.

After obtaining a 3D labeled cell mask – either directly from cellpose or after relabeling – cells smaller than the minimum size are removed. The minimum size is specified as a fraction of the “diameter” parameter used by the cellpose, e.g., if the cellpose “diameter” is 120 pixels and the fraction (“remove_small_diameter_fraction”) is 0.5, then all cells (or nuclei) with a diameter smaller than 60 pixels are removed. Technically, the removal is implemented by filtering the 3D labels by their volume, where the minimum volume is calculated from the minimum diameter using the sphere volume formula. In case of a very thin image (with only a few z-layers, as in the provided test examples), we provide an option of filtering by area rather than volume. For such thin images, the cell volume is not an adequate representation of cell size, since only a small fraction of the cell is contained in the image. In this case, we use minimum cell area (calculated as the circle area from the minimum diameter) as a criterion to filter cells based on their area averaged over all z-layers. The option to filter by volume or area is specified by the “remove_small_mode” parameters, which is set to either “3D” or “2D”.

Finally, we provide an option to remove cells at the image border, which is specified by the “clear_border” parameter (True or False). This option removes cells/nuclei that are touching the xy border of the image. The cell removal is not applied in z, since most of the cells touch image border in z due to the thin sample nature.

# **Puncta segmentation**:

The puncta segmentation procedure consists of the following steps: (1) Laplacian of Gaussian (LoG) filter to identify potential puncta centers; (2) filtering of the potential puncta centers by comparing their intensities to the background signal of the cell; (3) thresholding of the image either in the original intensity space or the LoG filtered space; (4) watershed segmentation of the thresholded image using the filtered puncta centers as seeds; (5) optionally, excluding puncta that are too big or that extend beyond the cell mask.

Step 1:
First, the candidate puncta centers are detected using the scikit-image implementation of the Laplacian of Gaussian (LoG) filter, which is a common blob detector. This implementation allows the user to specify the minimum and maximum size of the target puncta, number of size scales applied, option to remove overlapping centers, and a threshold to remove low intensity centers (specified in the LoG filter scale). All these parameters can be specified by the user and adjusted using the setup_puncta_analysis.ipynb notebook with immediate feedback on the result.

Step 2:
If the cell or nuclei segmentation masks are available, there is an option to additionally filter out puncta centers based on their intensities relative to the background intensity of the cells. The background intensity of each cell is calculated as a specified percentile (default is 50: the median) of the image intensity in the cell (of the channel in which the puncta are detected). If the “global_background” parameter is set to False, individual background values are used for each cell. Otherwise, the global background is calculated as a specified percentile (default is 95) of the background intensities of all cells in the image. Image intensity at each puncta center is compared to the background intensity, and the center is removed if its intensity is lower than the background multiplied by the “threshold_background” (specified by the user, default is 3).

Step 3:
Next, to segment the puncta from the background, the puncta channel is thresholded in either original intensity space or LoG space. The method of thresholding is specified by the “segmentation_mode” and “threshold_segmentation” parameters. The “segmentation_mode” takes value 0, 1, or 2 and determines the way the “threshold_segmentation” is applied. For mode 0, the image is thresholded in the LoG space, i.e., the result of the LoG filter is used for thresholding; the segmentation threshold is applied as the absolute value, i.e., all pixels with LoG intensity higher than the threshold are assigned to the foreground / puncta. For mode 1, the image is thresholded in the LoG space, as in mode 0, but a relative threshold is applied; for this, the background LoG signal is calculated in each cell similarly to step 2, and the LoG image is thresholded at the value of the “threshold_segmentation” multiplied by the background value. For mode 2, the thresholding is done for the raw fluorescent channel, and the threshold is calculated relative to the cell background signal calculated in step 2. The background for steps 1 and 2 is calculated globally and the same value is used for all cells (note: this is a different background than the one used in step 2 for filtering of puncta centers).

It should be noted that since modes 1 and 2 require background calculation, they should only be used if the cell / nuclei segmentation is available. The value of the “threshold_segmentation” parameter should be chosen depending on the value of the “segmentation_mode” parameter, since it has different meanings for different modes. Thus, for mode 0, the value should be very small and close to 0, either positive or negative; we recommend starting with values 0.001 – 0.003 and decrease or increase the value depending on whether more/bigger or fewer/smaller puncta should be detected. For modes 1, and 2 the “threshold segmentation” value specifies how much higher than the background the puncta intensity should be. For mode 2, a good value range to start with is 2 – 3. Since the LoG filer increases the image contrast between the puncta and the background, the threshold value for mode 1 should be much higher; we recommend choosing a value from the range 20 – 100.

Step 4:
After the puncta are thresholded from the background, we split touching puncta using distance transform watershed, seeded with the puncta centers from step 2. This allows splitting puncta that were identified as one region after thresholding in step 3.

Step 5:
Finally, we provide an option to exclude puncta that are too big or that extend beyond the cell mask. If the option “remove_out_of_roi” is set to True, the puncta and puncta parts that extend beyond the cell masks are removed. If a value for the maximum radius (maxrad_um) is provided, all puncta are removed that have a volume greater than the volume of the equivalent sphere calculated from the maximum radius.
